# Computational Drug Repurposing Based on a Recommendation System and Drug–Drug Functional Pathway Similarity

**DOI:** 10.3390/molecules27041404

**Published:** 2022-02-18

**Authors:** Mengting Shao, Leiming Jiang, Zhigang Meng, Jianzhen Xu

**Affiliations:** 1 Computational Systems Biology Laboratory, Department of Bioinformatics, Shantou University Medical College (SUMC), Shantou 515041, China; 19mtshao@stu.edu.cn (M.S.); 18lmjiang@stu.edu.cn (L.J.); 2 Department of Computer Science, College of Computer Engineering and Applied Mathematics, Changsha University, Changsha 410005, China

**Keywords:** drug functional similarity, drug repurposing, pathway activities, recommender system

## Abstract

Drug repurposing identifies new clinical indications for existing drugs. It can be used to overcome common problems associated with cancers, such as heterogeneity and resistance to established therapies, by rapidly adapting known drugs for new treatment. In this study, we utilized a recommendation system learning model to prioritize candidate cancer drugs. We designed a drug–drug pathway functional similarity by integrating multiple genetic and epigenetic alterations such as gene expression, copy number variation (CNV), and DNA methylation. When compared with other similarities, such as SMILES chemical structures and drug targets based on the protein–protein interaction network, our approach provided better interpretable models capturing drug response mechanisms. Furthermore, our approach can achieve comparable accuracy when evaluated with other learning models based on large public datasets (CCLE and GDSC). A case study about the Erlotinib and OSI-906 (Linsitinib) indicated that they have a synergistic effect to reduce the growth rate of tumors, which is an alternative targeted therapy option for patients. Taken together, our computational method characterized drug response from the viewpoint of a multi-omics pathway and systematically predicted candidate cancer drugs with similar therapeutic effects.

## 1. Introduction

Drug resistance is a major problem in cancer treatment where cancer cells escape from the effects of anticancer compounds and patients suffer from recurrence and more aggressive forms of cancer. Although the exact mechanism is still not completely understood, accumulated evidence indicates that resistance to cancer therapy is mediated by the complex interplay between several key factors, such as intratumoral heterogeneity, metabolic reprogramming, and cancer microenvironment [1]. Therefore, it is urgent to develop novel therapy options, such as the combination of known drugs, to combat cancer cells. However, the development and validation of new drugs are time-consuming, expensive, and often prone to failure. Studies have shown that each drug takes an average of 10–15 years and more than USD 2 billion to create; but, the success rate is less than 10% [2].

Drug repurposing is used to identify new uses of existing or research drugs that are beyond their original indications [3]. Classic examples include Minoxidil (initially used for hypertension; now for hair loss), Viagra (initially used for angina; now to treat erectile dysfunction and pulmonary hypertension), and Rituximab (initially used for chronic lymphocytic leukemia and rheumatoid arthritis; now for non-Hodgkin’s lymphoma) [4]. Recently, bromocriptine has been approved for the treatment of type 2 diabetes in the U.S. due to the new finding that it is also a central dopamine agonist [5]. Compared with traditional drug discovery, the repositioning of known drugs has the advantages of lower cost and higher safety; thus, they may demonstrate a new way to accelerate the drug development process [6].

Many computational repositioning methods have been developed. Some researchers exploited the structural similarity of drug molecules or shared protein targets, whereas others leveraged prior knowledge of drug, target and disease interaction, and introduced systems biology approaches for drug repurposing. For example, we and others have developed network-based methods for finding molecules inhibiting tumor progression [7,8,9]. In this study, we adopted a recommendation system-based framework to prioritize drugs. The recommender system is widely applied in e-commerce to predict users’ preferences for selling products. Recently, it has demonstrated great promise in various bioinformatics problems, such as predicting protein subcellular localization, RNA–protein interactions, and drug repurposing [10,11,12,13]. In this study, we combined various cancer multi-omics datasets, such as gene profiling, DNA methylation and CNV data, and enriched them into KEGG pathways. Then, based on these functional features, a recommender system is utilized to predict drug response. Our results indicated that this model can achieve both comparable accuracy and clear biological mechanisms when evaluated on two large cancer drug response datasets (CCLE and GDSC).

## 2. Results and Discussion

### 2.1. Workflow Overview

**Figure 1:**
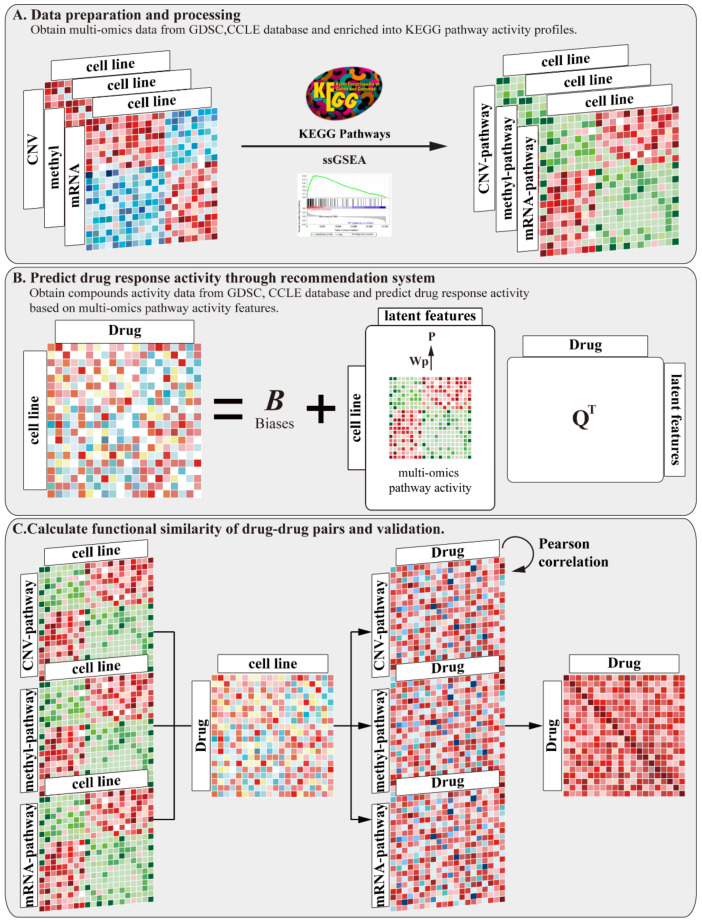
A pipeline to prioritize candidate compounds based on a drug functional similarity. Our method includes three main steps: (**A**) inferring multi-omics pathway activity profiles; (**B**) predicting drug response activity through recommendation system based on multi-omics pathway activity profiles; and (**C**) calculating drug–drug functional similarity and evaluating with other drug similarities to validate our result.

We developed a pipeline to prioritize candidate drugs based on a drug functional similarity. A flow diagram of the pipeline is shown in Figure 1. Our method consists of three main steps. (A) Download mRNA expression data, DNA methylation data, and copy number variation data from the GDSC and CCLE databases. Then, use single sample gene set enrichment analysis (ssGSEA) to project each type of omics data onto the KEGG pathway, thus obtaining multi-omics pathway activity profiles. (B) Download compounds’ activity data, and then predict the drug response activity through a recommendation system based on multi-omics pathway features. (C) Construct a drug functional similarity between the drugs by integrating the mRNA, methylation, and CNV pathway activity profiles and the predicted drug response activity. This functional similarity is compared with compound structural similarity and target distance similarity.

### 2.2. Evaluation of Predictive Drug Response Results

For evaluation, we compared the above pipeline (multi-omics pathway) with the following two scenarios: (1) only mRNA expression profiles are used to infer pathway activity, then feed the same recommendation system (mRNA-pathway); (2) the mRNA expression profile, but not pathway activity, is used directly in the same recommendation system (mRNA expression). NDCG is a generally accepted index for evaluating ranking recommendations. It ranges from 0 to 1, where 1 means that the model predicted the drug’s ranking accurately. As shown in Table 1, both the multi-omics pathway setting and mRNA-pathway setting are significantly better than the mRNA expression setting in both the GDSC and CCLE datasets. At the same time, these two scenarios also achieved a relatively smaller sum of squared error than when mRNA expression data were used, which indicated better robustness of the learning models.

This analysis suggests that pathway-based drug response prediction is better than gene-based prediction in the recommendation system. Furthermore, multi-omics pathway settings can achieve comparable accuracy with the single-omics (mRNA profile) pathway-based setting.

### 2.3. Drugs’ Effects on Biological Pathway Levels

Inspecting the multi-omics pathway activity profiles for each drug can reveal its functional effect in cells. In addition, comparing the multi-omics pathway activity profiles of each drug pair can elucidate their mechanistic similarity, which provides a functional understanding for their mutual replacement.

**Figure 2:**
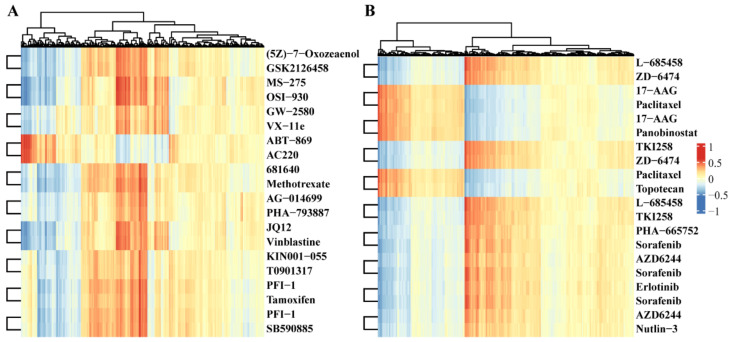
Heatmap of Pearson correlation coefficients of drugs in the activity of the multi-omics pathway. (**A**) Top 10 drug pairs based on functional similarity in GDSC database, in which each row represents a drug, and each column represents multi-omics pathways. The values are correlation coefficients. Drug names are listed at the right most columns. (**B**) Top 10 drug pairs based on functional similarity in the CCLE database.

The heatmap in Figure 2 shows the Pearson correlation of the top 10 drug repurposing pairs with the activity levels of multi-omics pathways. For example, (5Z)-7-Oxozeaenol and GSK2126458/omipalisib form a drug repurposing pair in GDSC datasets. Previous reports found that (5Z)-7-Oxozeaenol inhibits TAK1 [14] and GSK2126458 is the inhibitor of PI3K [15]. They have the highest correlation with “Toxoplasmosis pathway”, ”Tuberculosis pathway”, and ”Salmonells infection pathway” from the viewpoint of methylation, which may indicate that the two drugs have similar effects on the methylation of key genes in these pathways. In addition, for the L-685458 and ZD-6474/Vandetanib drug pair in the CCLE datasets, L-685458 is a kind of gamma-secretase inhibitor [16], and ZD--6474/Vandetanib efficiently suppresses RET kinase, including the vascular endothelial growth factor receptor and epidermal growth factor receptor signaling [17]. These two drugs both have the highest correlation with the “Bacterial invasion of epithelial cells pathway”, “ErbB signaling pathway”, and “Small cell lung cancer pathway” at the methylation level. Again, this result revealed that both L-685458 and ZD-6474 are involved in the methylation process of genes located in KEGG pathways.

Furthermore, these data also suggest that even though the drug targets are very different, their pathway activity in cells may have similar patterns, indicating that the drugs share common regulatory mechanisms. The activity pattern of the drug pairs across multi-omics pathways describes their functional similarity, which also lays the foundation for drug repurposing.

### 2.4. Comparison of Drug Pair Similarities

According to the activity patterns of the drug pairs across all the multi-omics pathways, we can assign each drug pair a functional similarity score. Table 2 lists the top 10 drug pairs’ similarities in the GDSC and CCLE datasets. For comparison, we also investigated the similarities based on the chemical structures and protein targets of these drug pairs in GDSC and CCLE datasets. As shown in Table 2, the functional similarity is higher than other similarities. These results suggest that drug responses at biological pathway levels are more likely to reflect their biological influences and explain why the two drugs can induce similar effects on the cell; i.e., it is not based on their molecular structure or the closeness of their protein targets in a PPI network, but rather due to their similar functional effects on the multiple-omics pathways. Furthermore, it confirmed that the functional similarity between drugs can prioritize their mutual replacement.

### 2.5. Case Study

The drug repurposing pair, Erlotinib and OSI-906, has a functional similarity of 0.978, indicating their similar therapeutic effects. Erlotinib potently blocks EGFR kinase activity and suppresses downstream signaling pathways, such as PI3K–AKT and MAPK [18]. In addition, these signaling pathways are promoted by other receptors, including IGF1R [19]. During embryonic development and postnatal growth, IGF1R is sensitized by ligands IGF-1 and -2 [20,21]. Researchers have indicated that the components of the IGF family are often abnormally expressed in cancers and activate tumorigenesis [20,22]. IGF1R overexpression is also associated with poor survival in some tumor types [23,24,25,26]. Furthermore, cancers have evolved a compensating mechanism for IGF1R inhibition [27]. A variant form of the insulin receptor (INSR-A) is induced by IGF-2 and insulin, which enhances proliferation and cell survival [28]. Thus, co-inhibition of IGF1R and INSR may provide enhanced antitumor activity [29,30].

Linsitinib (OSI-906) suppresses both IGF1R and INSR tyrosine kinase. Previous studies indicated it can inhibit proliferation in a variety of tumor cell lines and xenograft models [31,32]. Single-drug treatment of linsitinib in patients with solid tumors, such as melanoma and adrenocortical carcinoma, has proven its antitumor activity [33,34,35]. From drug mechanistic analysis, it can be seen that although Erlotinib and OSI-906 inhibit different protein targets, their influences in cells converge on the same downstream pathway. This explains why they can be mutually repurposed.

In addition, this repositioning drug pair also implies that the combined use of both drugs may be a good therapeutic option, especially for overcoming drug resistance. The literature review confirms this conclusion. The combined IGF1R/INSR and EGFR blockade has shown enhanced inhibition of common downstream signaling pathways, and suppressed resistance to single-receptor blockades [22,30,36]. Preclinical investigations among NSCLC, breast, pancreatic, and colorectal cancer patients have indicated that the combined administration of IGF1R/INSR and EGFR inhibitors lead to additive effects on tumor growth [37,38,39,40,41]. Furthermore, IGF-2 is induced in erlotinib-resistant tumors and small-molecule IGF1R TKI sensitized the tumors to the EGFR inhibitor [42].

Finally, we set the functional similarity of multi-omics pathways that are >0.95 as a threshold to select candidate repurposing drug pairs. In total, we obtained 1015 out of 31125 drug pairs in the GDSC dataset, and 53 pairs out of 276 pairs in the CCLE dataset (Supplementary Table S1).

## 3. Materials and Methods

### 3.1. Data Sources and Data Processing

#### 3.1.1. Chemical Compounds Activity Data

GDSC drug data: The drug activity data of 250 unique drugs in 904 cancer cell lines were collected from the GDSC database (http://www.cancerrxgene.org/gdsc1000/GDSC1000_WebResources/Home.html). This page provides several Excel sheets referred to in the Iorio et al. paper [43] as Supplementary Materials. Drug responses were provided as the natural logarithm of half the maximal inhibitory concentration in a data matrix (file:TableS4A.xlsx, tab “TableS4A-IC50s”), which we subsequently converted to negative logarithms with a base 10, ensuring that the drug activity levels are expressed as the negative log of the half-maximal inhibitory concentration [−log10(IC50)]. A higher value represents more drug sensitivity in a cell line.

CCLE drug data: CCLE drug sensitivity data in the form of IC50 values were extracted from the file “CCLE_NP24.2009_Drug_data_2015.02.24.csv”, which is available on the CCLE website [44] and includes IC50 data for 24 anti-cancer drugs. Afterwards, the negative number of logarithms with a base 10 of the IC50 values was calculated for the drug activity values. In total, there were 24 unique drugs in 402 cell lines that we used for our analyses.

#### 3.1.2. Multi-Omics Expression Data

GDSC multi-omics data: Raw cell line expression array data were downloaded from ArrayExpress (file: E-MTAB-3610) [43], in which data is measured by the Affymetrix Human Genome U219 Array chip, and after RMA normalization of R packages ‘affy’ [45], the expression data of each gene in each cell line were obtained. The methylation data were extracted from the average pre-processed β-values for each of all CpG islands (file: F2_METH_CELL_Data.txt), which were also available on the download portal. For the CpG islands, we used the GPL13534–11288 reference to match genes to the CpG islands in the dataset. The copy number variation data were extracted from PICNIC [46], and the absolute copy numbers were derived from the Affymetrix SNP6.0 array data (file: cnv_abs_copy_number_picnic_20191101.csv). Thus, we derived the estimated value of the copy number of each gene. For each omics profile of the GDSC database, we converted the cell names to cosmic IDs based on the annotation file of the GDSC cell lines.

CCLE multi-omics data: Omics data (expression, methylation, and copy number variation) describing the cancer cell lines were acquired via bulk download from the Cancer Cell Line Encyclopedia (CCLE) (https://portals.broadinstitute.org/ccle/data, access date: 2020/08/05) [47]. In agreement with the original publications [44,47], expression data were obtained through Affymetrix U133+2 arrays and processed to obtain gene-centric RMA-normalized mRNA expressions (file: CCLE_Expression_Entrez_2012-09-29.gct) [48]. Raw Affymetrix CEL files were converted to a single value for each probe set using the Robust Multi-Array Average (RMA) and normalized using quantile normalization. Methylation data were derived by quantifying CpG islands using Reduced Representation Bisulfite Sequencing (file: CCLE_RRBS_tss_CpG_clusters_20181022.txt.gz). Copy number variation (CNV) data were acquired from the Affymetrix SNP6.0 arrays (file: CCLE_copynumber_byGene_2013-12-03.txt.gz). Copy numbers were normalized by comparingto the most similar HapMap normal samples [49]. Segmentation of the normalized log2 (CN/2) ratios was achieved using the circular binary segmentation (CBS) algorithm [47,50]. For mRNA expression profiles with duplicate gene IDs, we aggregated their expression values by their means for our analysis, and for methylation profiles with duplicate gene IDs, we aggregated their values by sum.

### 3.2. Inferring Multi-Omics Pathway Activity Profiles

First, we obtained 250 selected gene sets (C2) of the KEGG pathway from the previous research [51] and then used single sample gene set enrichment analysis (ssGSEA) with R packages “GSVA” [52,53] against KEGG pathway data to convert mRNA expression data, methylation level data, and copy number variation data into a pathway activity profile, which was accomplished by a “single sample” extension of GSEA [54], defining an enrichment score based on the degree of absolute enrichment of a gene set in each sample within a given dataset.

For the given sample *S*, the expression values of gene *G* of size *NG* were firstly rank-normalized. Then, the empirical cumulative distribution functions (ECDF) of the genes were used to calculate an enrichment score of *ES (G, S)* by a sum (integration) of the difference between a weighted ECDF of the genes in the signature $P_{G}^{\omega }$ and the ECDF of the remaining genes $P_{NG}$ as follows, where α is set to 0.25.

(1) $$ES\left(G,S\right)\;=\overset{N}{\underset{i=1}{\sum }}[P_{G}^{\omega }\left(G,S,i\right)\;-\;P_{NG}\left(G,S,i\right)]$$

(2) $$P_{G}^{\omega }\left(G,S,i\right)\;=\sum_{rj\in G,j\leq i}\frac{{\left|rj\right|}^{\alpha }}{\sum_{rj\in G}{\left|rj\right|}^{\alpha }}$$

(3) $$P_{NG}\left(G,S,i\right)\;=\sum_{rj\notin G,j\leq i}\frac{i}{\left(N-N_{G}\right)}$$

This procedure differs from the classic GSEA procedure in that the gene list is ranked by absolute expression and the enrichment score is obtained by an integration of the difference between the ECDFs. For the GDSC dataset, 228 resultant mRNA pathways, 201 methyl pathways, and 227 CNV pathways were extracted. In total, 656 multi-omics pathways were obtained. In addition, 223 mRNA pathways, 220 methyl pathways, and 228 CNV pathways were extracted for the CCLE datasets with a total of 671 multi-omics pathways. Finally, these pathway activity patterns were used to calculate drug–pathway associations and the drug pathway level similarity.

### 3.3. Predict Drug Response Activity through Recommendation System Based on Multi-Omics Pathway Activity Profiles

Here, we modified the CADRRes framework to predict drug response. The previous report indicated that the CADRRes algorithm outperformed other existing methods including elastic net regression and random permutations [13]. The first step in CaDRReS is to define the cell line features. In contrast to the original calculation with gene expression information, here we calculated cell line features based on the activity of the multi-omics pathway, i.e., by using Pearson’s correlation to compute every pair of cell lines, while utilizing the multi-omics pathway activity profiles. In total, we obtained 904 and 402 cell line features for GDSC and CCLE, respectively.

Models were pre-trained and tested independently on both CCLE and GDSC to avoid biases toward either of the datasets [55,56]. Then, the matrix factorization was used to train drug sensitivity models, which were computed based on the equation:

(4) $$\hat{S_{ui}}=\mu +b_{i}^{Q}+b_{u}^{P}+q_{i}\cdot p_{u}=\mu +b_{i}^{Q}+b_{u}^{P}+q_{i}{\left(x_{u}W_{p}\right)}^{T}$$

where $\hat{S_{ui}}$ is the computed sensitivity score of cell line u to drug i; μ is the general mean drug response; $b_{i}^{Q}$ and $b_{u}^{P}$ are bias terms for drug i and cell line u, respectively; $q_{i},p_{u}\in R^{f}$ are vectors for drug i and cell line u in the f-dimensional latent space; and $W_{P}\in R^{d\times f}$ is a transformation matrix that projects cell line features $x_{u}\in R^{d}$ onto the latent space. The value of f was set at 10. The source code of CaDRReS can be downloaded at https://github.com/CSB5/CaDRReS. Here, we used the CaDRReS_train_and_test.py file to predict GDSC and CCLE drug response activity data.

Two indicators were used to evaluate the drug response predicted by the recommendation system. The normalized discounted cumulative gain (NDCG) is a generally accepted score for comparing recommendations. It was computed as follows:

(5) $$NDCG\left(\hat{r},s\right)\;=\frac{DCG\left(\hat{r},s\right)}{DCG\left(r,s\right)}$$

(6) $$DCG\left(\hat{r},s\right)\;=\sum_{i}\frac{2_{i}^{s}-1}{\log_{2}\hat{i}+1}$$

where $\hat{r}$ is the output rank of drugs tested on a cell line, s is the observed drug activity values, and r is the known rank of drugs based on the drug activity values. The numerator in the DCG is designed to give more weight to drugs with higher sensitivity scores, while the denominator takes precedence over predicting drugs with a higher rank. In addition, the “sum of squared error” loss function was defined as:

(7) $$L\left(\theta \right)\;=\frac{1}{2\left|K\right|}\sum_{u}\sum_{i}e_{ui}^{2}$$

(8) $$e_{ui}=s_{ui}-\hat{s_{ui}}$$

where $s_{ui}$ and $\hat{s_{ui}}$ are the observed and predicted activity for cell line u using drug i, respectively; $\theta =\;\left\{b_{i},b_{u},W_{p},q_{i}\right\}$; and $\left|K\right|$ is the number of drug activity values in the dataset.

### 3.4. Calculate the Functional Similarity of Drug–Drug Pairs Based on Multi-Omics Pathways Profiles

We first calculated the Pearson correlation between the multi-omics pathway activity profiles and the drug activity profiles predicted by the recommendation system across cell lines in GDSC and CCLE datasets, respectively:

(9) $$r=\frac{\sum_{u=1}^{n}\left(D_{u,i}-\bar{D_{i}}\right)\left(P_{u,j}-\bar{P_{j}}\right)}{\sqrt{\sum_{u=1}^{n}{\left(D_{u,i}-\bar{D_{i}}\right)}^{2}}\sqrt{\sum_{u=1}^{n}{\left(P_{u,j}-\bar{P_{j}}\right)}^{2}}}$$

where $D_{u,i}$ is the predicted drug activity profiles with drug i and cell lines u, and $P_{u,j}$ is the multi-omics pathway activity profiles in method 3.2 with pathway j. Then, for both drug i and pathway j, the correlation based on the same cell lines as u can be computed. The Pearson correlation coefficient reflects the association between the multi-omics pathway activity and the drug response activity.

Next, based on the above multi-omics pathway–drug correlation matrix, the Pearson correlation coefficient of each pair of drugs is calculated, which reflects the functional similarity of each pair of drugs at the level of the multi-omics pathways and describes the functional similarity between the multi-omics pathway activity patterns, in which drugs with higher functional similarity have a higher probability of being involved in related biological pathways and treating similar diseases.

### 3.5. Molecular Structural Similarities and Drug Target Similarities

In order to make a strict comparison with the other similarity of drugs, we calculated the two-dimensional structural similarity of the drugs and the similarity of the protein-protein interaction (PPI) distance of the drug targets. All similarity measures were finally normalized to be in the range (0, 1). Here, the two-dimensional structure data of the drug comes from the SMILES format of the PubChem database, which was downloaded from Pubchem [57,58]. The similarity score between two drug molecules is calculated according to the two-dimensional Tanimoto coefficient score [59], based on R packages “RxnSim” [60]. It extracts structural features and is then defined as the size of the intersection divided by the size of the union of the feature sets. The structural features determine absorption, distribution, metabolism, excretion, and toxicity properties, which ultimately affect the pharmacological activity of the drug molecule.

The target data come from The Drug Gene Interaction Database [61] and the PPI data were downloaded from the STRING database [62]. The distances between each pair of drug targets were computed using an all-pairs shortest paths algorithm, found on the human PPI network for drugs, associated with more than one gene, and the minimal distance between the associated genes was used. Then, the shortest distances were transformed to similarity values using the formula described in Perlman et al. [63]:$\;S\left(p,p^{\prime }\right)\;=Ae^{-bD\left(p,p^{\prime }\right)}$, where $S\left(p,p^{\prime }\right)$ is the similarity value between two proteins and $D\left(p,p^{\prime }\right)$ is the shortest path between them in the PPI network. Then, *A, b* was set to 0.9 and 1 according to Perlman et al. [63]. Self-similarity was assigned a value of 1.

## 4. Conclusions

The long and expensive drug discovery process needs to develop its machine learning method for drug repurposing. We integrate a variety of transcriptomic resources, methylation, and CNV data into a recommendation framework. Not only can it accurately predict drug response, but also provide an interpretable mechanism on drug effects at the same time. This is particularly useful in personal drug prescriptions in cancer care and/or combating drug resistance. In addition, accumulated evidence revealed that some cancers share common mechanisms and molecular pathogenesis with neurodegenerative diseases, such as Alzheimer’s disease (AD) [64,65]. Thus, this framework can be utilized as a computational tool for generating mechanistic hypotheses and drug replacement for both cancer and AD when the drug activity and multi-omics data are comprehensive.

## Figures and Tables

**Table 1:** Performance and robustness comparison on drug response.

	GDSC Multi-omics Pathway	GDSC mRNA-Pathway	GDSC mRNA Expression	CCLE Multi-omics Pathway	CCLE mRNA-Pathway	CCLE mRNA Expression
NDCG	0.815	0.816	0.381	0.976	0.978	0.798
Sum of squared error	1.176	1.173	3.540	0.728	0.662	2.633

**Table 2:** Top 10 drug pairs’ similarities in GDSC and CCLE datasets.

Database	Drug1	Drug2	Functional Sim	SMILES Sim	PPI Sim
GDSC	(5Z)-7-Oxozeaenol	GSK2126458	0.998365459	0.162790698	1.08E−70
GDSC	MS-275	OSI-930	0.998193161	0.362694301	3.30E−77
GDSC	GW-2580	VX-11e	0.997189963	0.242063492	3.71E−84
GDSC	ABT-869	AC220	0.996300765	0.257462687	0.9
GDSC	681640	Methotrexate	0.996231842	0.236734694	6.46E−66
GDSC	AG-014699	PHA-793887	0.996134942	0.223529412	5.37E−72
GDSC	JQ12	Vinblastine	0.995927571	-	1.54E−91
GDSC	KIN001-055	T0901317	0.99574713	0.161111111	4.90E−75
GDSC	PFI-1	Tamoxifen	0.995377083	0.208092486	2.38E−66
GDSC	PFI-1	SB590885	0.995238003	0.194029851	2.74E−83
CCLE	L-685458	ZD-6474	0.994678	0.11349	1.18E−67
CCLE	17-AAG	Paclitaxel	0.994097	0.272071	0.9
CCLE	17-AAG	Panobinostat	0.99304	0.075472	3.97E−71
CCLE	TKI258	ZD-6474	0.992772	0.227439	0.9
CCLE	Paclitaxel	Topotecan	0.992513	0.291483	0.9
CCLE	L-685458	TKI258	0.989722	0.129946	2.38E−66
CCLE	PHA-665752	Sorafenib	0.98863	0.157855	0.9
CCLE	AZD6244	Sorafenib	0.98829	0.169047	0.9
CCLE	Erlotinib	Sorafenib	0.987973	0.256565	0.9
CCLE	AZD6244	Nutlin-3	0.987127	0.137107	0.9

## Data Availability

Not applicable.

## Supplementary Materials

Table S1: Candidate drug repurposing pairs and their functional structural and target similarities. Candidate drug repurposing pairs are selected with a cutoff function similarity of >0.95.

## Sample Availability

Not available.
